# Streptozotocin induces alpha-2u globulin nephropathy in male rats during diabetic kidney disease

**DOI:** 10.1186/s12917-021-02814-z

**Published:** 2021-03-04

**Authors:** Kanchana Kengkoom, Wannee Angkhasirisap, Tapanee Kanjanapruthipong, Rongdej Tungtrakanpoung, Khwanchanok Tuentam, Naphatson Phansom, Sumate Ampawong

**Affiliations:** 1 Academic Service Division, National Laboratory Animal Center, Mahidol University, 999, Salaya, Puttamonthon, Nakorn Pathom, 73170 Thailand; 2 Department of Tropical Pathology, Faculty of Tropical Medicine, Mahidol University, 420/6, Ratchawithi Road, Ratchathewi, Bangkok, 10400 Thailand; 3 Department of Biology, Faculty of Science, Naresuan University, 99, Moo 9, Phitsanulok-NakornSawan Road, Phitsanulok, 65000 Thailand

**Keywords:** Alpha-2u globulin, Diabetic kidney disease, Histopathology, Nephropathy, Streptozotocin

## Abstract

**Background:**

Alpha-2u globulin nephropathy mainly shows toxicological pathology only in male rats induced by certain chemicals and drugs, such as levamisole (antiparasitic and anticancer drugs). Streptozotocin (STZ) is also an anticancer-antibiotic agent that has been used for decades to induce a diabetic kidney disease model in rodents. The purpose of this study is to determine if STZ causes alpha-2u globulin nephropathy in male rats during an advanced stage of diabetic kidney disease. Alpha-2u globulin nephropathy, water absorption and filtration capacities (via aquaporin [AQP]-1, − 2, − 4 and − 5) and mitochondrial function (through haloacid dehalogenase-like hydrolase domain-containing protein [HDHD]-3 and NADH-ubiquinone oxidoreductase 75 kDa subunit [NDUFS]-1 proteins) were examined in STZ-induced diabetic Wistar rat model.

**Results:**

More than 80% of severe clinical illness rats induced by STZ injection simultaneously exhibited alpha-2u globulin nephropathy with mitochondrial degeneration and filtration apparatus especially pedicels impairment. They also showed significantly upregulated AQP-1, − 2, − 4 and − 5, HDHD-3 and NDUFS-1 compared with those of the rats without alpha-2u globulin nephropathy.

**Conclusions:**

STZ-induced alpha-2u globulin nephropathy during diabetic kidney disease in association with deterioration of pedicels, renal tubular damage with adaptation and mitochondrial driven apoptosis.

## Background

Alpha-2u globulin nephropathy, a deposition of alpha-2u globulin (a lipocalin family protein with proteolytic and hydrolytic-resistant activities) in proximal tubule lysosomes, is an important toxicological syndrome that presents only in male rats and is relevant to nephropathy and renal neoplasia [1]. Furthermore, alpha-2u globulin is synthesised in the liver of male rats under multi-hormonal control, especially androgen [2]. Industrial or environmental chemicals and drugs have been reported to cause alpha-2u globulin nephropathy in male rats, including unleaded gasoline, 1,4-dichlorobenzene, pentachloroethane, synthetic jet fuel and diesel, fuel marine, levamisole and RG7129 (β-site amyloid binding protein) [1, 3–6]. Histopathological changes in rats with alpha-2u globulin nephropathy include hyaline droplet deposition in the cytoplasm and lumen of the proximal tubule, tubular degeneration and regeneration, tubular dilatation and parenchymal inflammation [6]. Presently, it is well documented that streptozotocin (STZ) injections have been used to induce hyperglycaemia in rodents, which leads to renal injury with similarities to human diabetic nephropathy. Renal pathology in STZ-induced hyperglycaemic rats mainly consists of glomerular hypertrophy, hypercellularity, tubular dilatation and atrophy, thickening of the glomerular basement membrane and mesangial expansion [7]. However, there have not yet been reports that alpha-2u globulin nephropathy can be induced by STZ injection in male rat as shown by previously mentioned drugs. Interestingly, Sun and colleague suggested that, in an STZ-induced diabetic rat, alpha-2u globulin and its modified form are dysregulated in renal mitochondria, leading to a reduction in β-oxidation of long chain fatty acids, decreased energy supply, increased fatty acid depositions and thus renal damage [8]. Along these lines of thought, histopathology, immunohistochemistry, electron microscopy and immunogold labelling techniques were performed to demonstrate the presence of alpha-2u globulin nephropathy in STZ-induced diabetic rats in relation to the alteration of (i) water reabsorption and filtration function as characterised by aquaporins (AQP)-1, − 2, − 4 and − 5, (ii) mitochondrial energetic maintenance protein using haloacid dehalogenase-like hydrolase domain-containing protein (HDHD)-3 and (iii) mitochondrial apoptotic marker by NADH-ubiquinone oxidoreductase 75 kDa subunit (NDUFS)-1. Clinico-histopathological and fine morphological appearances are also discussed.

## Results

### STZ induces alpha-2u globulin nephropathy in moribund hyperglycaemic rats

**Fig. 1:**
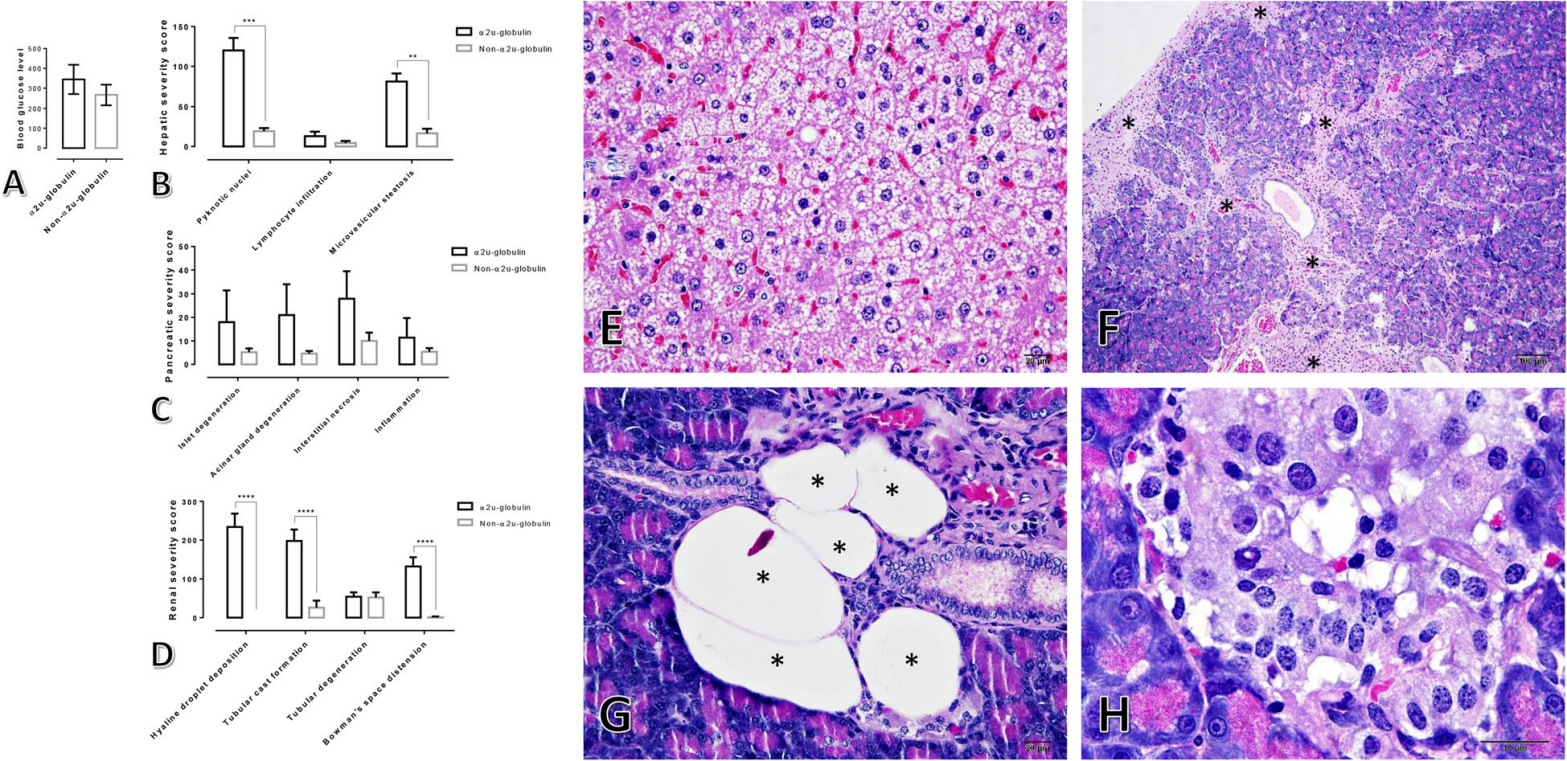
Blood glucose level and histopathological score from STZ-induced hyperglycaemic rats. **a-d**: Blood sugar level and histopathological changes in liver, pancreas and kidney using the H-score; **e-h**: histopathological appearance of the liver and pancreas by H&E staining; **e**: diffused centrilobular hepatic microvesicular steatosis; **f** (*): focal pancreatic necrosis, interstitial inflammation and fibrosis; **g** (*): focal cyst formation in the pancreatic gland; **h**: cellular swelling and degeneration in the Islet of Langerhans

**Fig. 2:**
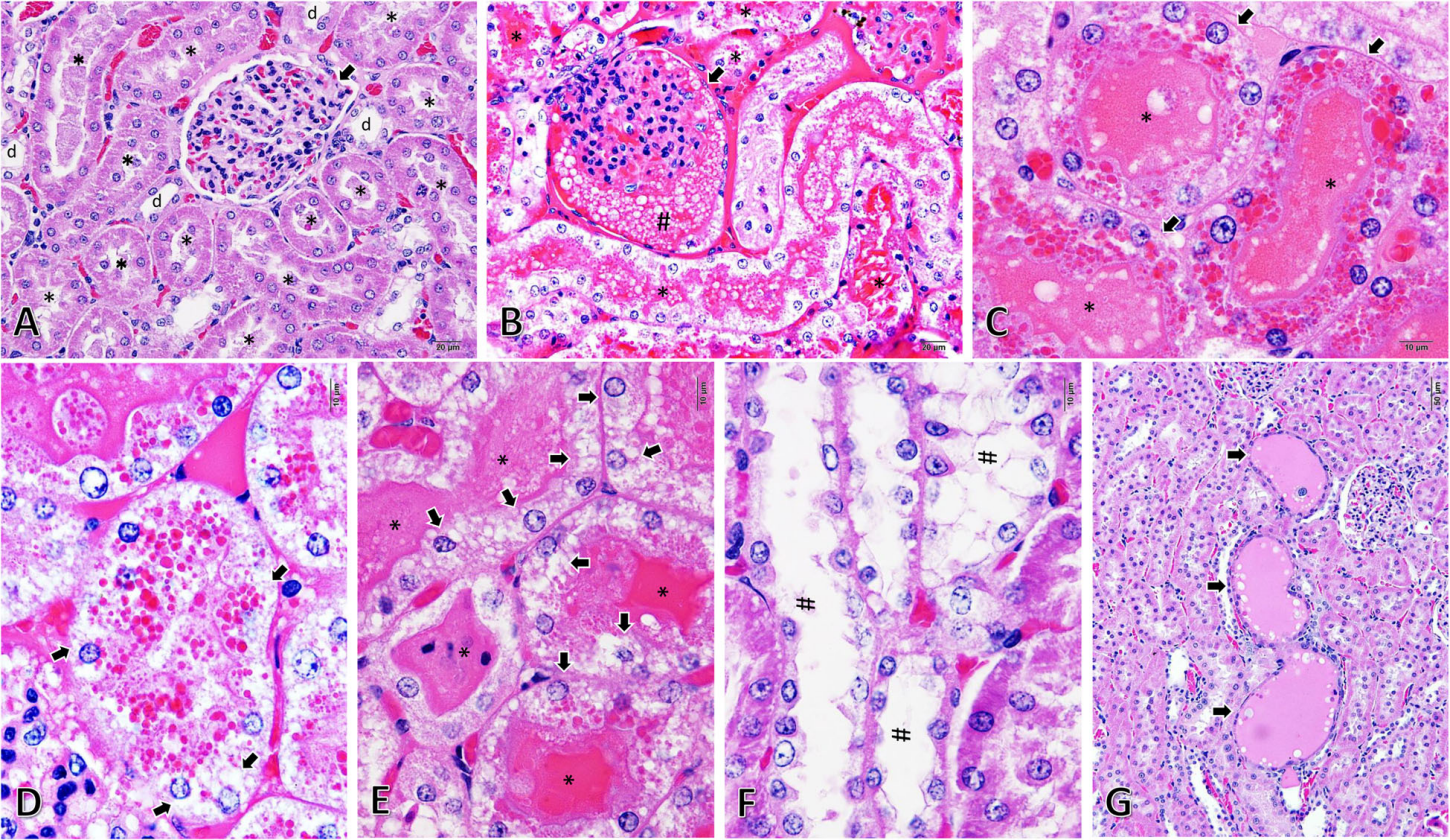
Histopathological changes in the kidneys of STZ-induced hyperglycaemic rats during diabetic kidney disease. **a-g**: Renal histopathological appearance by H&E staining; **a**: intact kidney from non-alpha 2u-globulin nephropathic rats with preserved PCTs (*), DCTs (d) and glomerulus (arrow); **b-g**: pathological changes in alpha 2u-globulin nephropathic rats consisting of Bowman’s space distension (**b**; arrow) with proteinaceous fluid deposition (**b**; **#**), PCT degeneration and hyaline cast deposition (**b**; *, **c**; *, **d**; arrow and **e**; *), intracytoplasmic hyaline droplet deposition in PCTs (**c**; arrow and **d**), vacuolated degeneration in the PCT (**d**; arrow) with sloughed epithelial cells (**e**; arrow) and tubular degeneration (**f**; #) and regeneration with flattening and basophilia (**g**; arrow)

As a consequence of STZ-induced hyperglycaemia in male rats, they all had a blood glucose level > 200 mg/dL (Fig. 1a) with polyuria 7 days post-induction. Twelve of them (12/20; 60%) showed severe clinical illness within 10–14 days post-induction, such as anorexia, depression, weight loss (≥20%) and moribund, while the rest had acceptable clinical manifestations with fair prognosis. Ten of the moribund rats (10/12; 83.33%) had obviously reddish urine and were microscopically diagnosed as having alpha 2u-globulin nephropathy. The blood sugar level between the rats with (*N* = 10) or without (*N* = 10) alpha 2u-globulin nephropathy was found to be not significantly different (Fig. 1a). Histopathological changes in the liver, pancreas and kidneys from the rats with and without alpha 2u-globulin nephropathy were scored as shown in the Fig. 1b–d, respectively. Although histopathological lesions of the pancreas in alpha 2u-globulin nephropathic rats tended to be higher than in the rats without alpha 2u-globulin nephropathy, the difference was not statistically significant (Fig. 1c). In addition, the alpha 2u-globulin nephropathic rats almost exhibited a significantly higher hepatic and renal histopathological scores than that presented in non-alpha 2u-globulin nephropathic rats, particularly pyknotic nuclei and microvesicular steatosis in the liver, and intracytoplasmic hyaline droplet deposition, tubular cast formation, and Bowman’s space distension (Fig. 1b,d). A hallmark of histopathological findings in alpha 2u-globulin nephropathy are the deposition of a hyaline cast in the urinary space and renal tubule, particularly in proximal convoluted tubules (PCTs) (Fig. 2b-e), distal convoluted tubules (DCTs) and collecting duct (CD), intracytoplasmic hyaline droplet deposition in the PCTs (Fig. 2c,d) and tubular degeneration with sloughed epithelial cells and regeneration (Fig. 2f,g). However, there is no evidence of renal neoplasia in this study.

### Renal ultrastructural changes in alpha-2u globulin nephropathy rats

**Fig. 3:**
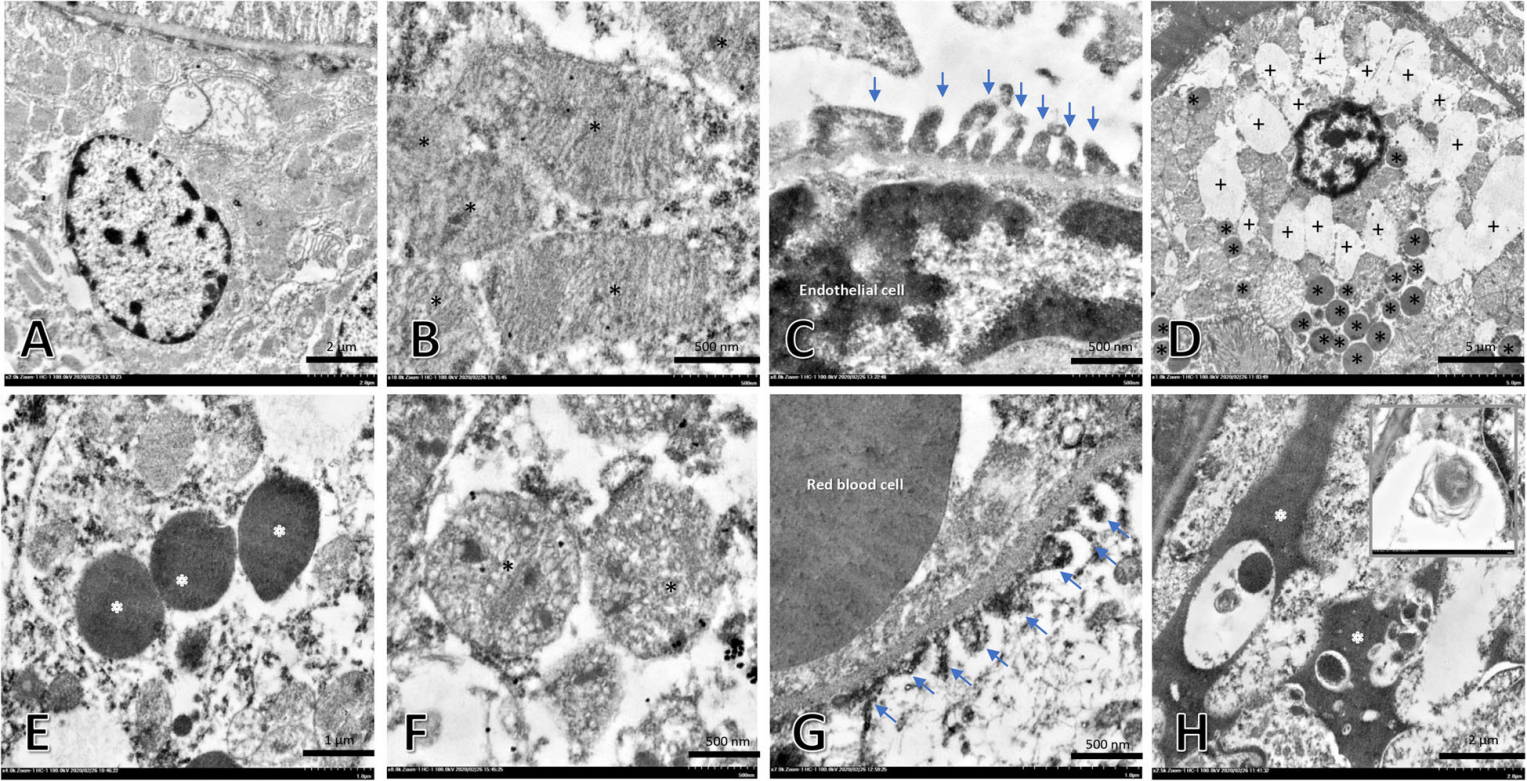
Ultrastructural changes in the kidney from STZ-induced hyperglycaemic rats with or without alpha 2u-globulin nephropathy. **a**: Epithelial cells in the PCT without alpha 2u-globulin deposition; **d,e**: epithelial cells in the PCT with alpha 2u-globulin deposition (*) and vacuolated degeneration (+), intact (**b**; *) and swelling or degenerative (**f**; *) mitochondria in the rats with (**f**) or without (**b**) alpha 2u-globulin nephropathy; **c,g**: podocyte foot processes (arrow) in the rats with (**g**) or without (**c**) alpha 2u-globulin nephropathy; **h**: hyaline cast (*) deposition in the urinary space; **h**-inset: higher magnification of the lamellated body

To characterise ultrastructural changes in the renal tubular mitochondria and renal filtrate apparatus including pedicels, glomerular basement membrane and endothelial cells, we conducted electron microscopy studies (Fig. 3a-h). Epithelial cells of the PCTs in non-alpha 2u-globulin nephropathic kidneys had better preserved architecture with less vacuolar degeneration (Fig. 3a) than those observed in alpha 2u-globulin nephropathic kidneys (Fig. 3d; +). Intracytoplasmic hyaline droplets were characterised by 0.5–3.0 μm of lobulated electron-dense material in the cytoplasm of epithelial cells of the PCT (Fig. 3d,e; *). Mitochondrial swelling and degeneration (Fig. 3f; *) were generally found in alpha 2u-globulin nephropathic kidneys compared to more intact mitochondria found in non-alpha 2u-globulin nephropathic kidneys (Fig. 3b; *). Moreover, pedicels of the podocyte showed severe degeneration in the alpha 2u-globulin nephropathic kidneys (Fig. 3g; arrow) compared with that of non-alpha 2u-globulin nephropathic kidneys (Fig. 3c; arrow). Hyaline cast deposition was also detected in the urinary space with lamellated debris material (Fig. 3h; *).

### AQP-1, − 2, − 4 and − 5 are upregulated in alpha-2u globulin nephropathy rats

**Fig. 4:**
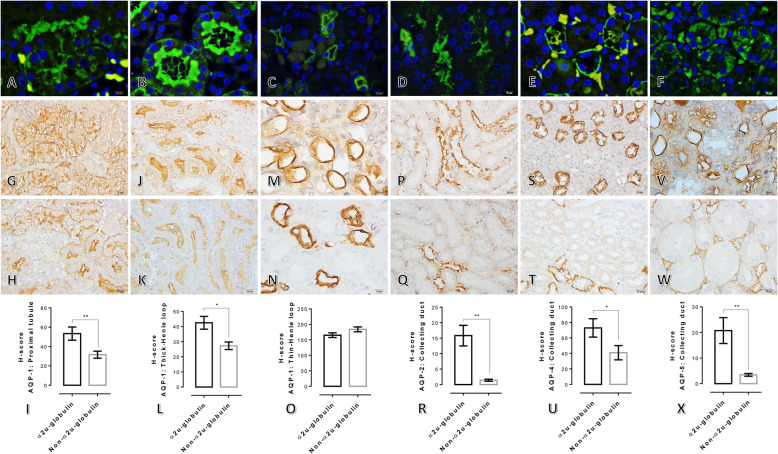
Renal AQP-1, − 2, − 4 and − 5 expression in rats with and without alpha 2u-globulin nephropathy. **a-f**: Immunofluorescence staining of AQP-1 in the PCT (**a**) and thick and thin segments of the Henle loop (**b** and **c**, respectively) and AQP-2, − 4 and − 5 in the CD (**d-f**, respectively) in rats with alpha 2u-globulin nephropathy; immunoreactivity is represented as green; DyLight 594; **g-x**: immunohistochemical staining of AQP-1 (**g-o**), − 2 (**p-r**), − 4 (**s-u**) and − 5 (**v-x**) in rats with (**g, j, m, p, s and v**) or without (**h, k, n, q, t** and **w**) alpha 2u-globulin nephropathy and their expression H-scores

To determine the expression of water channel membrane proteins under hyperglycaemia with or without alpha 2u-globulin nephropathy, immunohistochemical and immunofluorescence staining of AQPs was examined. The results revealed immunolocalisation of AQP-1 and -2, − 4, and − 5 in the PCTs and CD, respectively (Fig. 4a–f). AQP-1, − 2, − 4 and − 5 were found to be upregulated in the alpha 2u-globulin nephropathic kidneys as labelled on PCTs (Fig. 4g–o) and CD (Fig. 4p–x), respectively.

### Altered mitochondrial proteins in alpha-2u globulin nephropathy rats

**Fig. 5:**
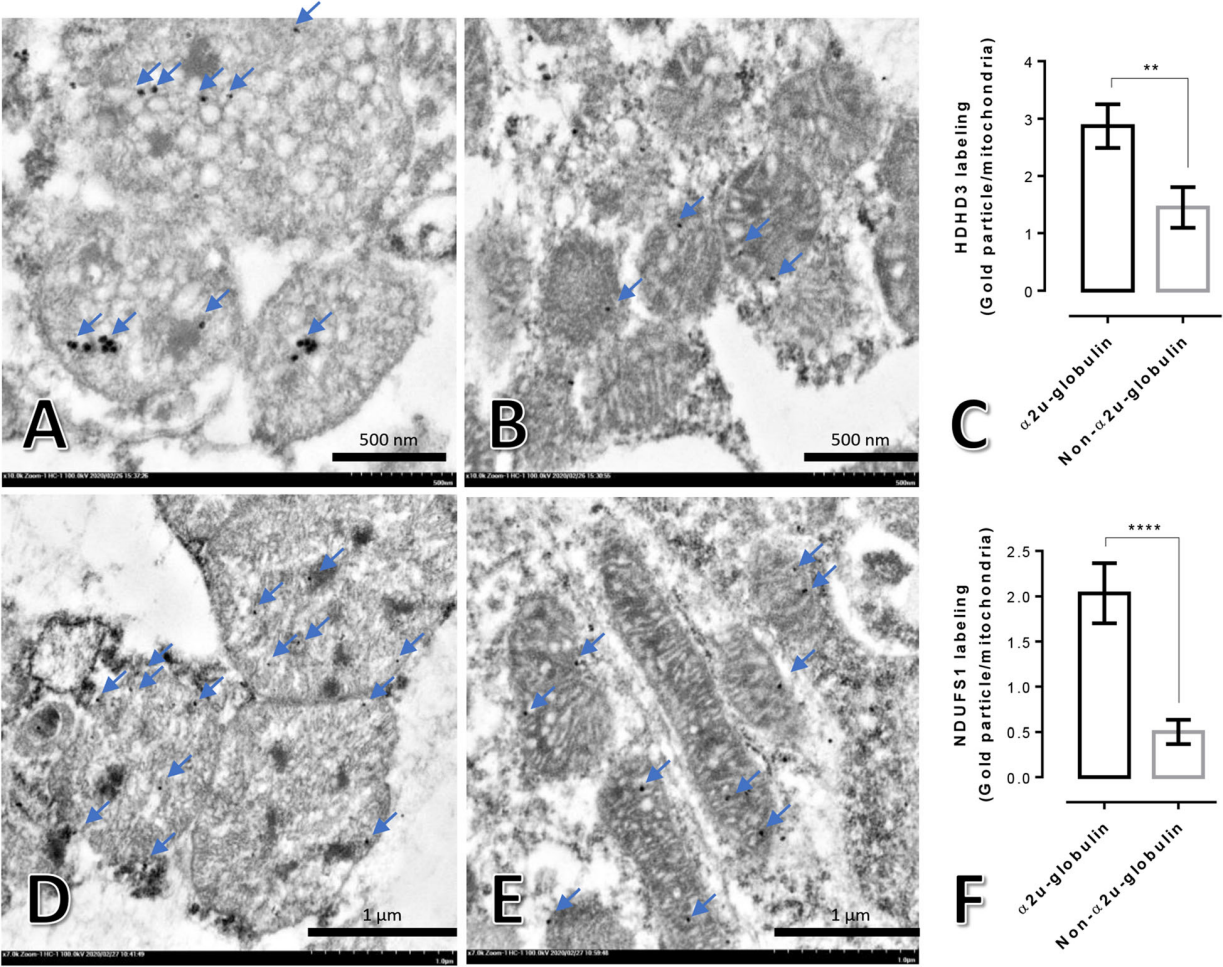
HDHD-3 and NDUFS-1 immunogold labelling in renal mitochondria from rats with and without alpha 2u-globulin nephropathy. **a-c**: gold labelling (arrow) of HDHD-3 in mitochondria from alpha 2u-globulin nephropathic rats (**a**) and non-alpha 2u-globulin nephropathic rats (**b**) and presented via a bar graph (**c**); **d-f**: gold labelling (arrow) of NDUFS-1 in mitochondria from alpha 2u-globulin nephropathic rats (**d**) and non-alpha 2u-globulin nephropathic rats (**e**) and presented via a bar graph (**f**)

Regarding to mitochondrial proteins maintaining for cellular integrity, mitochondrial HDHD-3 preserves both metabolic and energetic properties of cell [9]. In addition, NDUFS-1 acts as a critical caspase substrate to enhances cytochrome release and membrane permeabilization during apoptotic process [10]. It has been reported that he reduction of NDUFS-1 leads to mitochondrial damage and apoptosis [11]. To characterise mitochondrial function in terms of energetic balance and apoptosis via HDHD-3 and NDUFS-1 expression between the rats with or without alpha 2u-globulin nephropathy, immunogold labelling was performed. The results showed that the expression levels of HDHD-3 and NDUFS-1 were significantly increased in the mitochondria of PCTs from the alpha 2u-globulin nephropathic kidneys when compared with those of non-alpha 2u-globulin nephropathic kidneys (Fig. 5).

## Discussion

Sex- and species-specific diseases have been reported for decades since the discovery of unleaded gasoline was shown to lead to kidney tumours in male rats but not in females and both sexes of mice [3]. Likewise, the characterisation of alpha 2u-globulin nephropathy in male rats has also been discussed for decades [1–6, 8, 12, 13]. Several chemicals and drugs have been demonstrated to induce alpha 2u-globulin nephropathy only in mature male rats in association with neoplasia enhancement. Alpha 2u-globulin is synthesised in the liver under the influence of androgenic hormone and is released into blood circulation. This protein is freely filtered by the glomeruli and is reabsorbed by the S2 segment of the proximal tubule [3, 14–16] with persistent deposition due to its resistance to hydrolytic and proteolytic enzymes in the lysosomes, and approximately half of them are excreted in the urine [14, 15]. The accumulation of alpha 2u-globulin is cytotoxic and leads to single cell necrosis, a nidus for granular cast formation and reversible re-epithelialisation as presented by regenerative tubules [2, 17, 18]. Enhanced cellular proliferation initiates the transformation of proximal tubules to preneoplastic and neoplastic lesions [2, 19]. The primary histopathological change in alpha 2u-globulin nephropathy is intracytoplasmic “hyaline droplet” or “eosinophilic body” deposition in the proximal tubules with a variety of forms from spherical to polyangular [1, 4–6, 12, 13, 17, 19].

STZ, a nitrosourea alkylating agent or anticancer-antibiotic drug, has occasionally been used as a cytotoxic agent for treating some types of human tumours, e.g., lymphoma, sarcomas and Islet of Langerhans cancer [20]. It has also been extensively used for developing rodent models of diabetes and diabetic nephropathy. Interestingly, the present study demonstrates that during diabetic kidney disease induced by STZ exhibits alpha 2u-globulin nephropathy in > 80% of the moribund male rats. Levamisole is also an example of an anticancer and antiparasitic drug that causes alpha 2u-globulin nephropathy only in male rats. Similar to previous studies, intracytoplasmic hyaline droplet deposition in PCTs (Fig. 2c,d) leads to cellular degeneration as characterised by the increment in vacuolated degeneration (Fig. 2d,e) and tubular degeneration and regeneration (Fig. 2f,g, respectively). In contrast to other chemicals or drugs that induce 2u-globulin nephropathy, preneoplastic and neoplastic lesions were not observed in end-stage renal kidney disease induced by STZ. Moreover, electron micrographs also show the presence of mitochondrial degeneration and swelling in rats with alpha 2u-globulin nephropathy (Fig. 3d,e). These results clearly suggest that the cytotoxic properties of alpha 2u-globulin cause cellular and organelle damages. Additionally, considering the glomerular filtration capacity in alpha 2u-globulin nephropathic rats, this study demonstrates deterioration of the filtering apparatus, especially pedicels, as shown in the Fig. 3f,g. However, the detail mechanisms involved in this impairment caused by alpha 2u-globulin deposition require further study.

Diabetic nephropathy is a microangiopathic complication present in one-third of diabetes mellitus patients [21]. It has been claimed that dysregulation of the water channel membrane protein “aquaporin; AQP” in the kidney plays an important role in the pathogenesis of several kidney diseases including diabetic nephropathy [21–23]. Eight AQPs, AQP-1–7 and − 11, are expressed in the kidney to maintain normal urine concentration [23]. Several reports indicate that alterations of AQP-1, − 2, − 4 and − 5 expression are highly associated with renal diseases. AQP-1 functions in hypertonicity formation and is expressed in apical and basolateral membranes of proximal tubules, descending thin limbs of Henle and descending vasa recta [24]. It also localises in the β-laminin of the glomerular basement membrane [22]. AQP-2, a urine concentration regulator under anti-diuretic hormone, is located at the apical membrane of the collecting duct [25]. AQP-4, a water permeability regulator, is located at the basolateral membrane of the collecting duct and exports water into the cytoplasm via AQP-2 [23]. Lastly, AQP-5 is located in type B intercalated cells of the collecting duct with unclear function [26]. Upregulation of glomerular AQP-1 is found in all forms of human renal diseases, probably due to compensation for losing cellular integrity [22]. Upregulation of AQP-2 and -5 is closely related to the progression of diabetic nephropathy in diabetic patients and are good candidates to use for diagnosis [21, 27]. Recently, Go and Zhang also reported that an increase in AQP-5 in patients with diabetic nephropathy is independently associated with a reduction in the glomerular filtration rate [28]. In addition, a STZ-induced diabetic rat model exhibits a high level of anti-diuretic hormone, leading to upregulation of AQP-2 as a compensatory mechanism [29]. Dysregulation of intrarenal AQP-4 is involved in end-stage renal disease in HIV patients with glomerulosclerosis and renal tubular dysfunction [30]. In the present study, immunohistochemical studies reveal significant upregulation of AQP-1, − 2, − 4 and − 5 in the alpha 2u-globulin nephropathic rats (Fig. 4). These findings in relation to the increases in AQP-1, − 2, − 4 and − 5 responses are needed further studies to be proven for the involved specific mechanisms such as (i) are they likely compensatory during high cellular and mitochondrial degeneration due to alpha 2u-globulin deposition in the PCTs, (ii) are they associated with an advanced stage of diabetic kidney disease, (iii) are they related to a depletion of glomerular filtration capacity in association with the presence of pedicels disruption and (iv) are they correlated to renal tubule dysfunction, particularly PCTs, DCTs and CD.

According to mitochondrial function and its architecture, mitochondrial dysfunction is a crucial factor in the pathogenesis of diabetic kidney diseases regarding reactive oxygen species overproduction, apoptosis activation and mitophagy defects [31–37]. The kidney is an extreme oxygen consumption organ, which renders it sensitive to mitochondrial dysfunction. A hyperglycaemic environment also contributes to direct damage of renal tubular cells [31]. Dysregulation of essential mitochondrial genes in diabetic kidney diseases has been reported in relevance to the severity of renal pathology, e.g., glomerular endothelial injury, glomerulosclerosis and podocyte defects [33]. A change in the metabolic energy source under diabetic conditions results in increased oxygen consumption in the kidney and leads to renal hypoxia, ischaemia and necrosis [8, 32]. Our recent studies have demonstrated that cellular power synthesis (Haloacid Dehalogenase-Like Hydrolase Domain-Containing [HDHD]-3) and a mitochondrial apoptotic marker (NADH: ubiquinone oxidoreductase core subunit S1 [NDUFS-1]) in liver mitochondria in sericin-fed rats are preserved compared to those of non-treated rats under hypercholesterolemic conditions [38, 39]. In this study, the immunogold labelling technique indicates significant upregulation of HDHD-3 and NDUFS-1 in the alpha 2u-globulin nephropathic rats (Fig. 5). This suggests the high incidence of degenerative mitochondria in the alpha 2u-globulin nephropathic kidney, which attempt to increase energetic protein for the maintenance of renal function and integrity even when high levels of apoptosis were also observed.

## Conclusions

During diabetic kidney disease induced by STZ injection in male rats, alpha 2u-globulin nephropathy was predominately observed in association with upregulation of renal water channel membrane proteins (AQP-1, − 2, − 4 and − 5), mitochondrial energetic maintenance protein (HDHD-3) and mitochondrial apoptotic protein (NDUFS-1). All of these phenomena are likely due to compensation for renal damage in advanced stages of kidney disease. These findings are useful for understanding the pathogenesis of alpha 2u-globulin nephropathy in association with diabetic kidney disease induced by STZ infection.

## Methods

### STZ-induced hyperglycaemic rat model

Animal experimentation was conducted at the Research and Development Unit, Academic Service Division, National Laboratory Animal Center, Mahidol University (NLAC-MU). Animal experimentation was performed following to the Thai Animals for Scientific Purposes Act, B.E. 2558 and the Guidelines for the use of animals of the National Research Council of Thailand. Eight week-old male (*n* = 20) Wistar rats were obtained from NLAC-MU. All of the rats were housed in a temperature-, humidity- and illumination-controlled room and fed ad libitum with standard diet and reverse-osmosis water. Consequent to the acclimatisation period, all rats were fasted for 6 h before being intraperitoneally injected with a single dose of 45 mg/kg streptozotocin (Sigma-Aldrich, USA) in fresh 0.1 M citrate buffer, pH 4.0 to induce hyperglycaemia [37]. Fasting blood glucose was examined in all of the rats, and a blood sugar level of ≥200 mg/dL was considered diabetic stage. Clinical manifestations were carefully observed daily by trained personnel for 2 weeks. Then all rats were humanely euthanised using an overdose of carbon dioxide inhalation. However, in case of moribund rats or rats that lost ≥20% of their weight before 2 weeks were found, an early endpoint of those rats was done as mentioned above. Their kidneys were collected, divided into two and then fixed in 10% neutral buffer formalin and 2.5% glutaraldehyde in 0.1 M sucrose phosphate buffer (SPB) for histopathologic and electron microscopic studies, respectively.

### Histopathological studies

To demonstrate the presence of alpha 2u-globulin nephropathy in the STZ-induced diabetic rats and other histopathological changes in the liver and pancreas, histopathological studies were performed. Fixed kidneys, liver and pancreas underwent standard tissue processing and were cut into 5 μm thick sections. These sections were then stained with haematoxylin and eosin (H&E) and examined under a light microscope, focusing on (i) intracytoplasmic hyaline droplet deposition, tubular cast formation, tubular degeneration, tubular regeneration and Bowman’s space distension with proteinaceous fluid deposition for kidney, (ii) pyknotic nuclei, lymphocyte infiltration and centrilobular microvesicular steatosis for liver and (iii) Islet of Langerhans degeneration, vacuolar degeneration in the acinar gland and interstitial cell necrosis and inflammation of the pancreas. The lesions were semi-quantitatively graded using H-score (distribution [~ 0–100%/section] × severity score [0–3: 0 = absent, 1 = mild, 2 = moderate and 3 = severe]) as shown in our previous studies [39–43].

### Immunohistochemical and immunofluorescence studies

To determine the pathogenesis of alpha 2u-globulin nephropathy induced by STZ injection, as relevant to water absorption and glomerular filtrate function via AQP, immunohistochemical (IHC) and immunofluorescence (IF) studies were conducted using EnVision FLEX/HRP kit (DAKO, Denmark) and VectaFluor Duet immunofluorescence double labelling kit, DyLight 488 Anti-rabbit (green)/DyLight 594 Anti-mouse (red) (VECTOR, USA), respectively. The sections were deparaffinised in xylene, hydrated in a series of graded ethanol and heat-retrieved to enhance the antigenicity in citrate buffer, pH 6.0. Polyclonal rabbit anti-AQP-1, − 2, − 4 and − 5 (Millipore, USA) antibodies were incubated on the tissues. Appropriate secondary antibodies matching their conjugate and visualisation system from the kit were applied to the sections. The nuclei were counterstained by either haematoxylin or VECTASHIELD Antifade mounting medium with DAPI (VECTOR, USA) for IHC and IF, respectively. Immunolocalisation was measured using the H-score as mentioned above. In addition, the area of expression as a percentage was determined using an image analysis programme (ImageJ, version 1.51 J8, NIH). Briefly, five images of labelled areas were captured and transformed to binary images. Immunolocalisation was defined by the threshold mode and determined as an area fraction (%).

### Electron microscopic studies

To demonstrate the fine morphological structure of alpha 2u-globulin nephropathy in STZ-induced diabetes, electron microscopic studies were performed. The kidneys were again fixed with 1% osmium tetroxide in 0.1 M SPB, dehydrated in a series of graded ethanol, infiltrated and embedded in LR white resin (EMS, USA), polymerised in a 65 °C oven for 24–48 h, cut into 100 nm thickness and finally stained with uranyl acetate and lead citrate. Ultrastructural changes in relation to alpha 2u-globulin nephropathy were examined under a transmission electron microscope (TEM) (Hitachi; model HT7700, Japan).

### Immunogold labelling technique

To clarify the immunolocalisation of HDHD-3 (energetic maintenance protein) and NDUFS-1 (apoptotic protein) in the renal mitochondria, immunogold labelling technique were used. After the sections were blocked with 50 mM glycine and 5% bovine serum albumin (BSA) (EMS, USA), they were incubated with the described primary antibodies for 1 h at room temperature. Immunoglobulin (Ig) G conjugated with 10 nm gold particles (EMS, USA) was then applied to the sections for 1 h. Silver enhancement was performed using the Aurion R-Gent SE-EM kit (EMS, USA). Finally, the sections were stained with lead citrate and uranyl acetate and examined under TEM, focusing on the amount of gold labelling/mitochondria. Fifty mitochondria/group were assessed.

### Statistical analysis

GraphPad PRISM, version 6.05, was used for statistical analysis. Either independent *t*-tests or analysis of variance was performed to characterise the difference between the groups and was expressed as the mean ± SEM. The 95% confidence interval *p* < 0.05 was considered statistically significant.

## Data Availability

The datasets used and/or analysed during the current study are available from the corresponding author upon reasonable request.

## Abbreviations

AQP: Aquaporin
BSA: Bovine serum albumin
CD: Colleting duct
DCTs: Distal convoluted tubules
H&E: Haematoxylin and eosin
HDHD: Haloacid dehalogenase-like hydrolase domain-containing protein
IF: Immunofluorescence
IHC: Immunohistochemical
NADH: Nicotinamide adenine dinucleotide
NDUFS: NADH-ubiquinone oxidoreductase 75 kDa subunit
PCTs: Proximal convoluted tubules
SPB: Sucrose phosphate buffer
STZ: Streptozotocin
TEM: Transmission electron microscope
